# Internal Jugular Vein Cross-Sectional Area Enlargement Is Associated with Aging in Healthy Individuals

**DOI:** 10.1371/journal.pone.0149532

**Published:** 2016-02-19

**Authors:** Christopher Magnano, Pavel Belov, Jacqueline Krawiecki, Jesper Hagemeier, Clive Beggs, Robert Zivadinov

**Affiliations:** 1 Buffalo Neuroimaging Analysis Center, Department of Neurology, School of Medicine and Biomedical Sciences, University at Buffalo, Buffalo, NY, United States of America; 2 MRI Clinical and Translational Research Center, School of Medicine and Biomedical Sciences, University at Buffalo, Buffalo, NY, United States of America; 3 Centre for Infection Control and Biophysics, University of Bradford, Richmond Road, Bradford BD7 1DP, United Kingdom; 4 Institute for Sport, Physical Activity and Leisure, Leeds Beckett University, Leeds, LS1 3HE, United Kingdom; Instituto Cajal-CSIC, SPAIN

## Abstract

**Background:**

Internal jugular vein (IJV) narrowing has been implicated in central nervous system pathologies, however normal physiological age- and gender-related IJV variance in healthy individuals (HIs) has not been adequately assessed.

**Objectives:**

We assessed the relationship between IJV cross-sectional area (CSA) and aging.

**Materials and Methods:**

This study involved 193 HIs (63 males and 130 females) who received 2-dimensional magnetic resonance venography at 3T. The minimum CSA of the IJVs at cervical levels C2/C3, C4, C5/C6, and C7/T1 was obtained using a semi-automated contouring-thresholding technique. Subjects were grouped by decade. Pearson and partial correlation (controlled for cardiovascular risk factors, including hypertension, heart disease, smoking and body mass index) and analysis of variance analyses were used, with paired t-tests comparing side differences.

**Results:**

Mean right IJV CSA ranges were: in males, 41.6 mm^2^ (C2/C3) to 82.0 mm^2^ (C7/T1); in females, 38.0 mm^2^ (C2/C3) to 62.3 mm^2^ (C7/T1), while the equivalent left side ranges were: in males, 28.0 mm^2^ (C2/C3) to 52.2 mm^2^ (C7/T1); in females, 27.2 mm^2^ (C2/C3) to 47.8 mm^2^ (C7/T1). The CSA of the right IJVs was significantly larger (p<0.001) than the left at all cervical levels. Controlling for cardiovascular risk factors, the correlation between age and IJV CSA was more robust in males than in the females for all cervical levels.

**Conclusions:**

In HIs age, gender, hand side and cervical location all affect IJV CSA. These findings suggest that any definition of IJV stenosis needs to account for these factors.

## Introduction

In recent years a number of studies have reportedly linked internal jugular vein (IJV) anomalies to a range of different central nervous system diseases and aging including multiple sclerosis (MS), [1, 2] Parkinson's disease, [3] Meniere’s disease, [4, 5] and Alzheimer’s disease. [6–8] The IJVs play an important role in the cerebral venous drainage system, [9, 10] and constriction of these vessels has the potential to increase the hydraulic resistance of the venous pathways back to the heart. [11] However, due to a paucity of available data regarding the structural and physiological behavior of the IJVs in healthy individuals (HIs), it is unknown whether or not the reported IJV anomalies/stenoses are pathological in nature or just normal physiological variants related to gender, location and side. [12] Furthermore, the extent to which the morphology of the IJVs changes with aging has not been fully characterized. Consequently, there is a clear need to robustly measure and characterize the IJVs of HIs at all ages, so that distinctions between what is typical and atypical can be made with greater confidence.

Previous studies have assessed IJV cross-sectional area (CSA) using a variety of imaging techniques, including catheter venography, [13–15] Doppler ultrasound, [1, 2] computed tomography, [16] and magnetic resonance venography (MRV) [17–19] However, these studies have primarily investigated the IJVs CSA in the context of pathologies such as MS, rather than established the structural and physiological behavior of HIs. Furthermore, they have generally not considered the impact of aging on IJV CSA or controlled for cardiovascular risk factors.

Against this background, we investigated left and right IJV CSA variance at levels C2/C3, C4, C5/C6 and C7/T1 in 193 HIs of various ages. The aim of the study was to characterize the morphology of the IJVs at these cervical levels and to evaluate how CSA changes with respect to age, gender, cervical location and hand side.

## Materials and Methods

### Subjects and clinical data

This study utilized baseline data from an ongoing prospective study of cardiovascular, environmental and genetic risk factors in MS that enrolled over 1,000 subjects with MS, HIs and other neurologic diseases. [2, 20] The inclusion criteria for this sub-study were: a) age range 10 to 80 years old, b) being HI c) having an MRV exam performed within 30 days of physical/neurologic examination with the standardized study protocol. Subjects were required to meet the health screening requirements on physical and neurologic examination. Subjects also needed to complete a health screening questionnaire containing information about medical history (illnesses, surgeries, medications, etc.). History of known vascular abnormalities, presence of systemic or neurologic (cerebrovascular or neurodegenerative disease, positive history of alcohol abuse, etc) and pregnancy precluded enrollment in the study. Recruited subjects included hospital personnel, local advertisement respondents, and spouses/relatives of patients receiving clinical care at our center.

Participants underwent a clinical and MRV examination. All subjects were assessed with a structured environmental questionnaire, and had a physical and neurological examination. Cardiovascular risk factors were collected from all participants in-person by a trained interviewer with cross-examination of medical records. [20]

The study was approved by the University of Buffalo Institutional Review Board and written informed consent was obtained from all subjects. In case of subjects under 18 years old, the written consent was obtained by their caretakers, as approved by University of Buffalo Institutional Review Board.

### MRV acquisition

All subjects were examined on a GE 3.0T Signa Excite HD 12.0 Twin Speed 8-channel scanner (General Electric, GE, Milwaukee, WI) with a maximum slew rate of 150T/m/s and maximum gradient amplitude in each orthogonal plane. A 2-dimensional MRV sequence was acquired for all IJV CSA measurements. The MRV obtained 150, 1.5 mm-thick slices using a 320x192 matrix (frequency x phase) with a 22.0 cm field of view (FOV) and a phase field of view (pFOV) of 75% for a resolution of 0.69 x 1.15 x 1.5 mm^3^. Additional imaging parameters included Echo Time (TE) / Repetition Time (TR) / Flip Angle (FA) of 4.3 ms / 14 ms / 70°, and a Bandwidth (BW) of 31.25 kHz, for a total acquisition time of 5:19. MRV was acquired in a “true” (non-obliqued) axial orientation with one average, and no parallel imaging techniques were employed.

### MRV analyses

#### Cross-sectional area analysis

IJV assessment was performed using CSA region of interest (ROI) analysis on the 2D MRV with the Java Image Manipulation Tool (JIM) version 5.0 (http://www.xinapse.com), at specific cervical locations. The sequence was viewed orthogonally to assess which slices corresponded to the desired anatomical coverage, namely C2/C3, C4, C5/C6, and C7/T1. Within each of these locations, the operator determined the slice on which the IJV had a minimum CSA, and then used the ROI Toolkit to select the right and left IJVs. An example case, with location selection on an orthogonal (coronal) view of the MRA, and corresponding IJV CSA ROIs is shown in S1 Fig. To best select the edges, we used the Contour ROI tool, part of the automated Preview Contours toolbox. When necessary, the operator manually adjusted the ROI boundary.

#### Reproducibility

Reproducibility was assessed using two raters performing IJV CSA analysis on a set of 25 MRVs twice, with analyses a minimum of 2 weeks apart. Raters were blinded to each other’s ROI assessments, as well as to their own prior set of ROIs. Intra- and inter-rater reproducibility was assessed using the Intra-class Correlation (ICC), with corresponding p- and q-values.

#### Statistical analysis

Statistical analyses were performed using the Statistical Package for Social Sciences (IBM Inc, version 21.0). The demographic and clinical differences were tested using Student’s t-test and chi-square tests. Paired samples t-tests were used to compare IJV sides, with analysis of covariance used to evaluate differences between the various age groups (the subjects were grouped by decade). Pearson correlation analysis explored association of age and IJV CSA, while the effect of individual and multiple cardiovascular risk factors (hypertension, heart disease, smoking and body mass index) on age and IJV CSA was explored in the partial correlation analysis. Due to multiple comparisons, a nominal p-value <0.01 was considered statistically significant using a two-tailed test.

## Results

### Demographic characteristics

The demographic and cardiovascular risk factors characteristics are presented in Table 1. The average age of the male subjects was 43 years and there were no significant difference between males and females (p = 0.21). There was no significant difference in distribution of HIs across age groups: <20 (n = 20), 20–29 (n = 38), 30–39 (n = 24), 40–49 (n = 30), 50–59 (n = 40), 60–69 (n = 29) and >70 (n = 12). Comparison of the cardiovascular risk factors showed no significant differences between the male and female subjects with respect to smoking, hypertension, heart disease and body mass index (Table 1).

**Table 1:** Demographic and cardiovascular risk factors characteristics of healthy individuals.

		All HIs (n = 193)	Males (n = 63)	Females (n = 130)	p value
**Overall age in years, mean (SD)**		43 (17.5)	40.7 (17.1)	43.9 (17.7)	0.21
	**<20 years**	16.1 (20)	17.4 (8)	15.3 (12)	0.17
	**20–29 years**	24.4 (38)	24.5 (13)	24.4 (25)	0.91
	**30–39 years**	33.8 (24)	34.2 (11)	33.5 (13)	0.61
	**40–49 years**	45.2 (30)	44.4 (7)	45.4 (23)	0.44
	**50–59 years**	53.3 (40)	53.6 (14)	53.2 (26)	0.70
	**60–69 years**	63.7 (29)	64.4 (7)	63.5 (22)	0.46
	**>70 years**	72.9 (12)	72.3 (3)	73.1 (9)	0.54
**Heart disease, n (%)**		20 (12.3%)	5 (10.6%)	15 (12.4%)	0.77	
**Hypertension, n (%)**		19 (11.2%)	6 (12.2%)	13 (10.8%)	0.79	
**Smoking, n (%)**		58 (32.2%)	16 (29.6%)	42 (33.3%)	0.63	
**BMI (kg/m**^**2**^**), mean (SD)**		26.8 (5.7)	27.6 (4.1)	26.5 (6.2)	0.37	

HIs–healthy individuals; SD–standard deviation; BMI–body mass index. P values were calculated using Student’s t-test for age and BMI and chi-square tests for heart disease, hypertension and smoking.

Intra- and inter-rater IJV CSA reproducibility: A high degree of inter- and intra-rater reproducibility was observed (S1 Table), with strong ICC values (p<0.001) found at all cervical levels between the raters. At all cervical levels, intra-rater reproducibility was found to be more robust than inter-rater reproducibility.

### IJV CSA hand side differences

The results for the mean left and right IJV CSAs at each cervical level are presented in Table 2. At all cervical levels the IJV CSA was significantly larger on the right compared to the left hand side (p<0.001) both for males and females. When the subjects were grouped by age, it was found for all age groups that the right IJV CSA was significantly larger than the left at all cervical levels (data not shown).

**Table 2:** Internal jugular vein cross-sectional area at different cervical levels shown according to gender and hand side.

	All HIs (n = 193)			Males (n = 63)			Females (n = 130)			p values					
	Total	Left	Right	Total	Left	Right	Total	Left	Right	TotalM v. F	LeftM v. F	RightM v. F	TotalL v. R	ML v. R	FL v. R
**C7/T1**	118.0 (79.3)	49.3 (37.5)	68.7 (53.7)	134.2 (81.2)	52.2 (40.2)	82.0 (54.2)	110.1 (77.4)	47.8 (36.2)	62.3 (52.4)	.*047*	.453	.*016*	**< .001**	**< .001**	**.001**
**C5 /C6**	97.4 (60.2)	42.1 (30.6)	55.4 (38.0)	106.3 (65.3)	43.7 (33.1)	62.6 (41.8)	93.1 (57.4)	41.3 (29.5)	51.9 (35.6)	.155	.603	.066	**< .001**	**< .001**	**< .001**
**C4**	91.3 (41.0)	38.8 (23.5)	52.5 (28.2)	101.5 (44.8)	42.0 (26.0)	59.5 (31.7)	86.3 (38.3)	37.2 (22.0)	49.0 (25.7)	.*016*	.188	.*016*	**< .001**	**< .001**	**< .001**
**C2/C3**	66.7 (31.4)	27.5 (18.0)	39.2 (24.1)	69.6 (33.3)	28.0 (19.0)	41.6 (25.4)	65.2 (30.5)	27.2 (17.6)	38.0 (23.5)	.364	.789	.298	**< .001**	**.001**	**< .001**

HIs–healthy individuals; M–males; F–females; SD–standard deviation. Values given are as mean (SD) in millimeter square (mm^2^). Paired t-test was used for left vs. right IJV CSA comparisons and unpaired t-test was used to test gender differences. P values < 0.01 are displayed ain bold and p values <0.05 in *italics*.

### IJV CSA location differences

**Fig 1:**
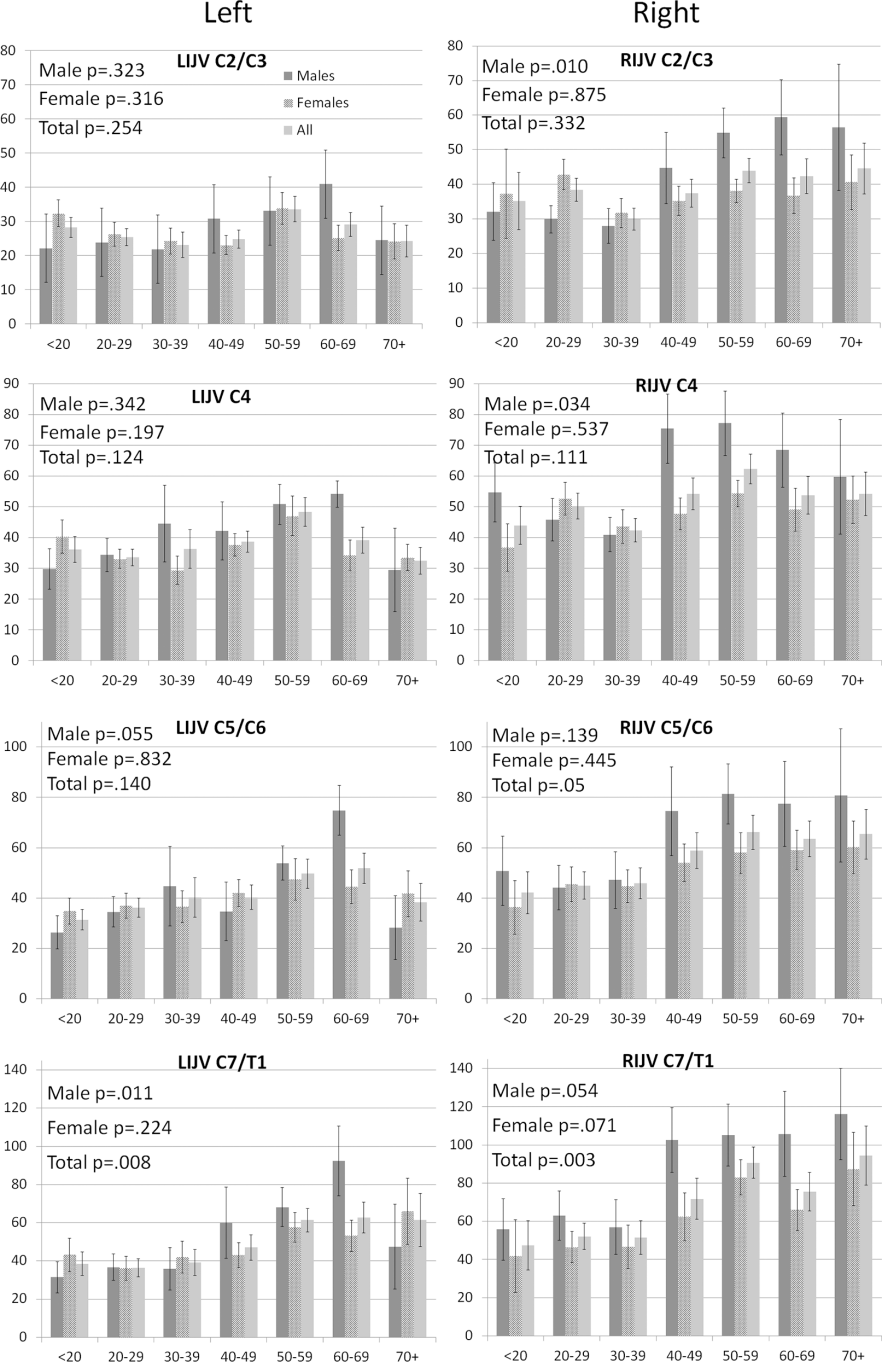
Internal jugular vein (IJV) cross-sectional area (CSA) vein according to hand side, cervical location and gender displayed by age groups. CSA is shown in mm^2^. Error bars represent standard error of the mean. P values were calculated with analysis of variance. Legend: LIJV–left internal jugular vein; RIJV–right internal jugular vein.

**Fig 2:**
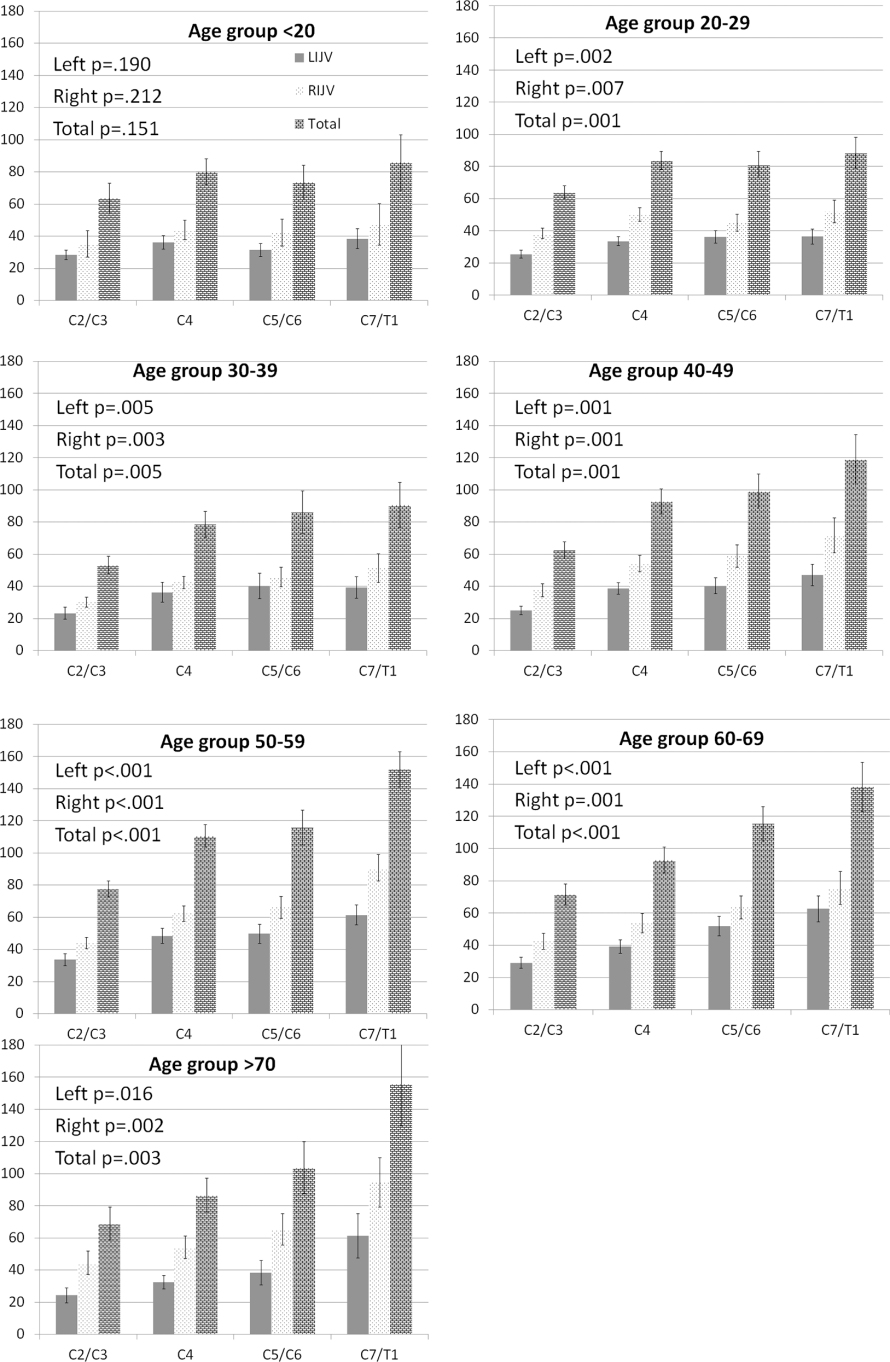
Internal jugular vein (IJV) cross-sectional area (CSA) vein according to age group hand side and gender displayed by cervical location. CSA is shown in mm2. Error bars represent standard error of the mean. P values were calculated with analysis of variance. Legend: LIJV–left internal jugular vein; RIJV–right internal jugular vein.

The results in Table 2 and Fig 1 show that IJV CSA was larger at the lower cervical levels (toward the heart), than at upper cervical levels (toward the head). This observation was true for both the left and right IJV CSAs for both gender, and was particularly pronounced in the older age groups (>40 years old), with the IJV CSA at C7/T1 being considerably larger than at the C2/C4 cervical level (Fig 2). With the exception of HIs <20 years of age, the larger IJV CSA at the lower cervical levels was significant for all age groups (Fig 2).

### IJV CSA gender differences

The IJV CSA in the females was generally smaller than those in the males (Table 2 and Figs 1 and 2), with statistical trends observed for the right IJV at cervical levels C4 and C7/T1. The difference in IJV CSA between the male and female HIs was more pronounced on the right hand side compared with the left.

### IJV CSA age differences

Fig 1 shows a general trend towards larger IJV CSA with increased age, something that was most visible at cervical level C7/T1 (left IJV, p = 0.008; right IJV, p = 0.003). This was more pronounced in male subjects for the right IJV CSA (C2/C3, p = 0.01; C4, p = 0.034; C7/T1, p = 0.054). Noticeably, the right IJV CSA was larger in HIs >40 years old, particularly in males compared to females (Fig 2).

The results shown in Fig 1 were corroborated by the correlation results shown in Table 3, which found a positive association between increased age and larger IJV CSA. The results were similar using the Pearson correlation or partial correlation analyses, adjusted for individual or multiple cardiovascular risk factors.

**Table 3:** Pearson and partial correlation analysis between age and internal jugular vein cross-sectional area according to cervical location.

		C7/T1			C5/C6			C4			C2/C3		
		LIJV	RIJV	Tot	LIJV	RIJV	Tot	LIJV	RIJV	Tot	LIJV	RIJV	Tot
No covariates	All	.28***	.28***	.33***	.17*	.24***	.28***	.09	.15*	.22*	.06	.14	.22*
	Males	.44***	.42**	.50***	.31*	.37**	.39**	.24	.32**	.37**	.26*	.46***	.50***
	Females	.21*	.24**	.26**	.11	.19*	.17	.03	.09	.08	-.04	-.02	-.03
All CVR factor as covariates	All	.24**	.26**	.29***	.19*	.29***	.28**	.14	.19*	.22**	.09	.16*	.17*
	Males	.49**	.45**	.54***	.42**	.56***	.57***	.44**	.48**	.59***	.47**	.62***	.72***
	Females	.15	.21*	.21*	.09	.18	.16	.01	.08	.06	-.05	-.02	-.05
Heart disease as covariate	All	.27**	.38***	**.38** ***	.15	.40***	.33***	.09	.36***	.30**	.07	.26**	.24*
	Males	.49***	.52***	.59***	.40***	.55***	.54***	.31*	.51**	.54***	.30*	.65***	.67***
	Females	.17	.24*	.24*	.10	.22*	.18	.04	.13	.11	-.04	-.01	-.03
Hypertension as covariate	All	.27**	.38***	.39***	.16	.40***	.34***	.09	.37***	.31**	.07	.27**	.25
	Males	.50***	.53***	.59***	.37*	.55***	.54***	.28	.51***	.53***	.29	.64***	.66***
	Females	.16	.24*	.24*	.010	.22*	.19*	.04	.13	.10	-.04	-.01	-.03
Smoking as covariate	All	.26**	.39***	.39***	.15	.40***	.33***	.08	.37***	.30**	.05	.28**	.24**
	Males	.47***	.45***	.50***	.35**	.41**	.44**	.28*	.36**	.44**	.28*	.51***	.54***
	Females	.18*	.24**	.25**	.09	.19*	.16	.03	.12	.10	-.07	-.01	-.05
BMI as covariate	All	.22*	.35***	.33**	.01	.38***	.30**	.03	.34**	.25*	.02	.26**	.21*
	Males	.47***	.45**	.52***	.35**	.43**	.45**	.27	.36**	.41**	.28*	.52***	.55***
	Females	.19*	.25**	.26**	.01	.20*	.18	.03	.12	.01	-.07	-.02	-.06

LIJV–left internal jugular vein; RIJV–right internal jugular vein; Tot–total; CVR–cardiovascular risk; BMI–body mass index.

The analyses were performed using Pearson correlation (no covariates) and partial correlation (using covariates) analyses. Covariates included individual and multiple cardiovascular risk factors (hypertension, heart disease, smoking and body mass index).

*** p <0.001

** p <0.01

* p <0.05. P values < 0.01 were considered significant **(bold)**, and less than 0.05 were considered trends *(italics)*.

Table 3 shows somewhat different correlation results between age and IJV CSA, according to gender. Older males exhibited stronger association with larger IJV CSAs than the females, with this effect being particularly evident for the upper cervical levels. Interestingly, after controlling for individual or multiple cardiovascular risk factors, the association between age and IJV CSA became more robust at all cervical levels for males, while tended to become weaker for females.

## Discussion

The main finding of the study, after adjusting for presence of cardiovascular risk factors, is that IJV CSA enlarges with aging, a phenomenon which is more pronounced in the right IJV than the left IJV, and in males than females.

IJVs are thin-walled floppy vessels that readily respond to changes in blood pressure. Any increase in IJV CSA will therefore be indicative of raised venous blood pressure and vessel distension, something that is consistent with impaired venous outflow, [21] possibly associated with increased intrathoracic pressure. [22, 23] Increased body mass index is positively associated with larger IJV CSA in the lower neck of both MS patients and HIs. [24] Because increased body mass index is linked with raised intra-abdominal pressure, [25] we hypothesize that it may inhibit cerebral venous drainage, [26] resulting in the IJV CSA enlargement. Given that body mass index tends to increases with age, it is perhaps not surprising that we observed a trend towards increased IJV CSA in the older age groups, something that was particularly marked in the right IJV of those who were >40 years of age. However, the association between increased IJV CSA and age, became even more robust after controlling for body mass index in males, while remained similar in females.

The marked gender difference, which was an unexpected finding, is difficult to explain. In the present study, both the males and females exhibited similar cardiovascular risk factors, suggesting that the aging and IJV CSA relationship may be related to other factors that can mediate these effects differently in male and females. For example, the discovery of endothelial progenitor cells has generated considerable interest in the field of vascular biology. These cells which arise from a population of circulating mononuclear cells have the capacity to form new blood vessels and contribute to vascular repair. [27] The circulating endothelial progenitor cell levels are reduced in patients with higher prevalence of cardiovascular risk factors. However, it has been shown that higher levels of estrogens in females during reproductive age is related to higher endothelial progenitor cell levels and consequently lower endothelial dysfunction. [28] Therefore, during the reproductive age, unique angiogenic properties of the female reproductive system, including female sex hormones, angiogenic growth factors, and stem cell regulatory molecules may contribute to explain observed differences in the current study. [29] Significant association between age and IJV CSA were only observed at C7/T1 cervical level in the females, whereas in the males, significant relationships were observed at all cervical levels. This suggests that age effects are more robust in males than females, particularly at upper cervical levels. Interestingly, when adjusting for cardiovascular risk factors (hypertension, heart disease, smoking and body mass index), we observed a differential effect in the males compared to females, with the associations becoming more robust in males, while remaining similar in females. Most importantly, adjusting for these covariates did not mask the effect of age in either the males or females.

It has been shown in HIs that blood flow in the IJVs is strongly influenced by the thoracic pump, with cerebral venous drainage greatly increased during deep inspiration. [30] The cumulative blood flow increases in the IJVs as they descend towards the thorax, with the consequence of IJV CSA tending to be greatest at the lower cervical levels, just as we observed in our study. As previously shown using Doppler ultrasound, [31] we also found the CSA of the right IJV was substantially larger than that of the left IJV for both gender, confirming the dominance of the right IJV in the HIs of all ages. However, it is noticeable that age effects were more robust in the right than the left IJV, and particularly in the males. While the reasons for this are unclear, it may be that being intrinsically larger vessels, the right IJVs have more capacity to distend compared with their counterparts on the left.

The research on the association between cerebral venous outflow and neurological disease have traditionally been focused on the concept of venous stenosis. [2, 17, 32] Historically, venous stenosis has been assessed by maintaining the threshold criteria used when assessing arterial stenosis, namely ≥50% narrowing of the vessels. [15, 32–36] A threshold of a CSA <0.3 cm^2^ on Doppler ultrasound has been recently proposed, [32] while other researchers have advocated for a threshold of 25 mm^2^ at C7/T1 or C5-C6, and 12.5 mm^2^ at C2/C3 cervical levels. [17–19, 37] Researchers have tended to apply these diagnostic thresholds in a uniform manner, making no allowances for the age and gender of subjects, or indeed differences between the size of left and right IJV CSA. [15, 17, 18, 32–36] The findings from this study suggest that this uniform approach may be flawed. For example, if the existing criteria were applied to the subjects in the current study, then a disproportionately high number of younger subjects would be considered stenotic. While it is beyond the scope of this paper to propose new diagnostic threshold criteria for IJV stenosis, our findings suggest that there is an urgent need to develop stenotic threshold criteria which will take into account subject age and gender, as well as distinguishing between the left and right IJV CSA at different cervical locations.

While our findings appear robust, as demonstrated by the high degree of intra- and inter-rater reproducibility, it is important to note that we only investigated the IJV CSA and neglected any collateral veins. It has been suggested that collateral veins play a compensatory role in the extracranial venous drainage system, [12] and further work will therefore be required to investigate this subject. The HIs enrolled in this study were part of the baseline data from an ongoing prospective study of cardiovascular, environmental and genetic risk factors in MS. [2, 20] Because the prevalence of MS is higher in females, our HI cohort was skewed toward more females than males, which is an important limitation of this study. Therefore it is necessary to confirm our findings in larger sample of male subjects. Another limitation of this study is that included relatively small number of subjects >70 years old, and older spectrum of HIs. Conversely, the inclusion of additional younger subjects would offer more information on IJV CSA in developing bodies. Therefore, future studies should include larger number of healthy subjects in age groups <20 and >70 years old to confirm our preliminary findings. Nevertheless, the current study is one of the largest IJV CSA studies in HIs reported in the literature. For example, a recent study measured IJV CSA at three neck levels comparing 2D TOF-MRV and dynamic 3D contrast-enhanced MRV in 40 HIs, [19] while another one assessed stenosis and flow of IJVs at the C2/C3 and C5/C6 levels in 67 HIs. [37]

In conclusion, we have shown that age, gender, hand side, and cervical location all affect IJV CSA. This suggests that when assessing patients for abnormalities of the cerebral venous drainage system, it is important to take in to account these factors, if erroneous diagnostic decisions are to be avoided.

## Supporting Information

S1 Fig Coronal view of orthogonal maximum intensity projection of the 2D axial magnetic resonance venography, allowing for visualization of the internal jugular veins (IJVs) from cervical levels C2 to T1. Region of interest slices were selected with the minimum cross-sectional area of the IJVs at different cervical locations (C2/C3, C4, C5/C6, and C7/T1).
(TIF)

S1 File IJV-CSA-Aging File. The minimal data set are within the paper and its supporting information files.
(TXT)

S1 Table Intra- and inter-rater reproducibility of internal jugular vein cross-sectional area in 25 healthy individuals at different cervical levels. (DOC)
