# Supplementary figures and images for: Internal Jugular Vein Cross-Sectional Area Enlargement Is Associated with Aging in Healthy Individuals
Source: PLoS One. 2016 Feb 19;11(2):e0149532. doi: 10.1371/journal.pone.0149532 (PMC4760933; doi:10.1371/journal.pone.0149532)

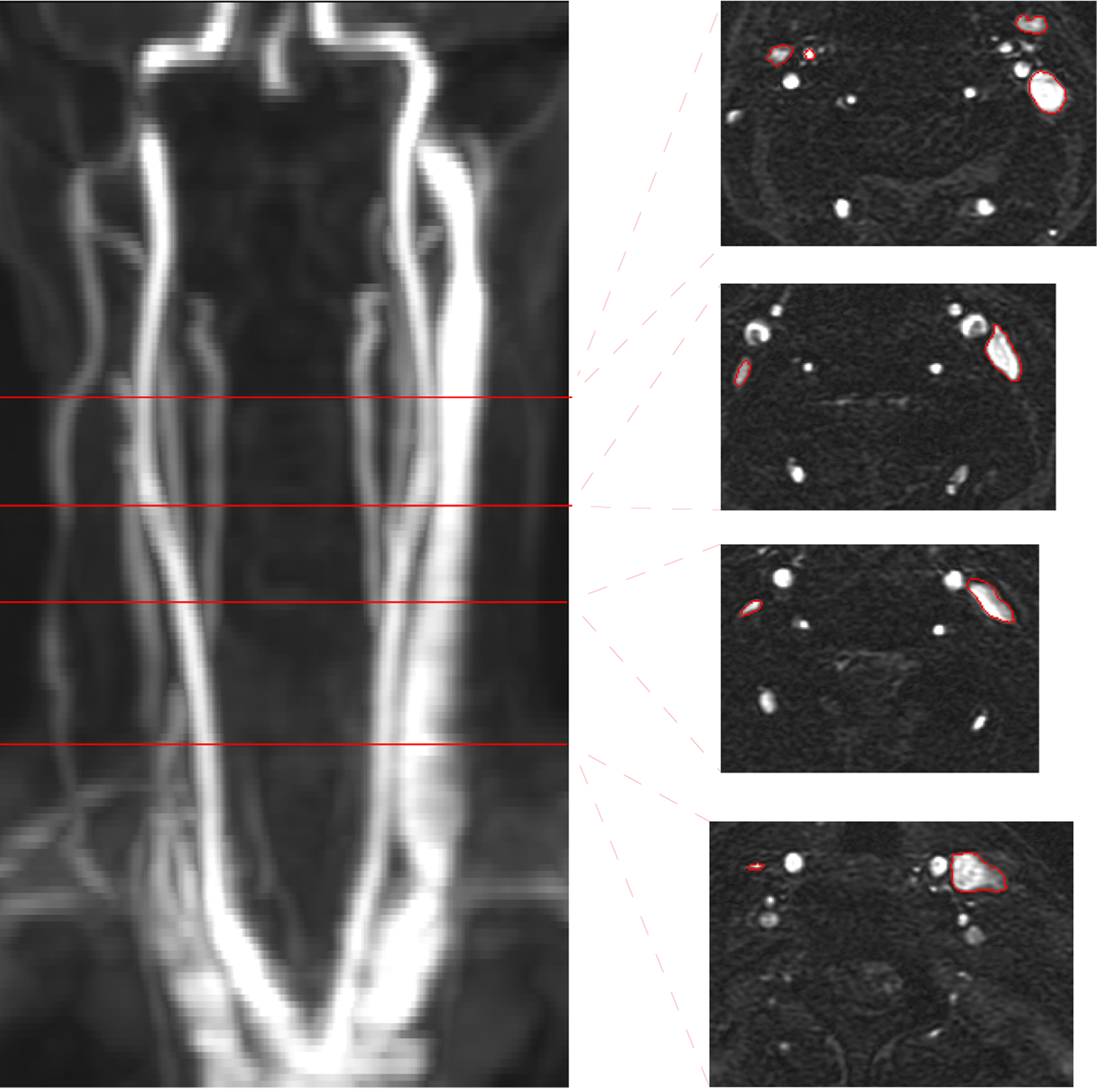

Supplement: S1 Fig — Region of interest slices were selected with the minimum cross-sectional area of the IJVs at different cervical locations (C2/C3, C4, C5/C6, and C7/T1). (TIF) [file pone.0149532.s001.tif]
