# Supplementary material for: Internal Jugular Vein Cross-Sectional Area Enlargement Is Associated with Aging in Healthy Individuals
Source: PLoS One. 2016 Feb 19;11(2):e0149532. doi: 10.1371/journal.pone.0149532 (PMC4760933; doi:10.1371/journal.pone.0149532)
Supplement: S1 Table — (DOC) [file pone.0149532.s003.doc]

|  | |  |  |  |  |
| --- | --- | --- | --- | --- | --- |
|  | |  |  |  |  |
|  |  |  |  |  |  |
|  |  |  |  |  |  |
|  |  |  |  |  |  |
|  |  |  |  |  |  |
|  |  |  |  |  |  |
|  |  |  |  |  |  |
|  |  |  |  |  |  |
|  | |  |  |  |  |
|  | |  |  |  |  |
|  | |  |  |  |  |
|  | |  |  |  |  |
|  | | | | | |

|  | **All HIs (n=193)** | | | **Males (n=63)** | | | **Females (n=130)** | | | **p values** | | | | | |
| --- | --- | --- | --- | --- | --- | --- | --- | --- | --- | --- | --- | --- | --- | --- | --- |
|  | Total | Left | Right | Total | Left | Right | Total | Left | Right | Total  M v. F | Left  M v. F | Right  M v. F | Total  L v. R | M  L v. R | F  L v. R |
| **C7/T1** | 118.0 (79.3) | 49.3 (37.5) | 68.7 (53.7) | 134.2 (81.2) | 52.2 (40.2) | 82.0 (54.2) | 110.1 (77.4) | 47.8 (36.2) | 62.3 (52.4) | *.047* | .453 | *.016* | **<.001** | **<.001** | **.001** |
| **C5 /C6** | 97.4 (60.2) | 42.1 (30.6) | 55.4 (38.0) | 106.3 (65.3) | 43.7 (33.1) | 62.6 (41.8) | 93.1 (57.4) | 41.3 (29.5) | 51.9 (35.6) | .155 | .603 | .066 | **<.001** | **<.001** | **<.001** |
| **C4** | 91.3 (41.0) | 38.8 (23.5) | 52.5 (28.2) | 101.5 (44.8) | 42.0 (26.0) | 59.5 (31.7) | 86.3 (38.3) | 37.2 (22.0) | 49.0 (25.7) | *.016* | .188 | *.016* | **<.001** | **<.001** | **<.001** |
| **C2/C3** | 66.7 (31.4) | 27.5 (18.0) | 39.2 (24.1) | 69.6 (33.3) | 28.0 (19.0) | 41.6 (25.4) | 65.2 (30.5) | 27.2 (17.6) | 38.0 (23.5) | .364 | .789 | .298 | **<.001** | **.001** | **<.001** |
|  | | | | | | | | | | | | | | | |

|  |  |  | | |  | | |  | | |  | | |
| --- | --- | --- | --- | --- | --- | --- | --- | --- | --- | --- | --- | --- | --- |
|  |  |  |  |  |  |  |  |  |  |  |  |
|  |  |  |  |  |  |  |  |  |  |  |  |  |  |
|  |  |  |  |  |  |  |  |  |  |  |  |  |
|  |  |  |  |  |  |  |  |  |  |  |  |  |
|  |  |  |  |  |  |  |  |  |  |  |  |  |  |
|  |  |  |  |  |  |  |  |  |  |  |  |  |
|  |  |  |  |  |  |  |  |  |  |  |  |  |
|  |  |  |  |  |  |  |  |  |  |  |  |  |  |
|  |  |  |  |  |  |  |  |  |  |  |  |  |  |
|  |  |  |  |  |  |  |  |  |  |  |  |  |  |
|  |  |  |  |  |  |  |  |  |  |  |  |  |  |
|  |  |  |  |  |  |  |  |  |  |  |  |  |  |
|  |  |  |  |  |  |  |  |  |  |  |  |  |  |
|  |  |  |  |  |  |  |  |  |  |  |  |  |  |
|  |  |  |  |  |  |  |  |  |  |  |  |  |  |
|  |  |  |  |  |  |  |  |  |  |  |  |  |  |
|  |  |  |  |  |  |  |  |  |  |  |  |  |  |
|  |  |  |  |  |  |  |  |  |  |  |  |  |  |
|  |  |  |  |  |  |  |  |  |  |  |  |  |  |
|  | | | | | | | | | | | | | |

**Supplement Table (S1 Table.)** Intra- and inter-rater reproducibility of internal jugular vein cross-sectional area in 25 healthy individuals at different cervical levels.

| **Level/Vessel** | **Inter-rater ICC (p, q)** | **Intra-rater ICC (p, q)** |
| --- | --- | --- |
| **C7/T1 RIJV** | 0.782 (<0.001, <0.001) | 0.999 (<0.001, <0.001) |
| **C7/T1 LIJV** | 0.691 (<0.001, <0.001) | 0.840 (<0.001, <0.001) |
| **C5/C6 RIJV** | 0.817 (<0.001, <0.001) | 0.986 (<0.001, <0.001) |
| **C5/C6 LIJV** | 0.722 (<0.001, 0.001) | 0.953 (<0.001, <0.001) |
| **C4 RIJV** | 0.863 (<0.001, <0.001) | 0.978 (<0.001, <0.001) |
| **C4 LIJV** | 0.881 (<0.001, <0.001) | 0.907 (<0.001, <0.001) |
| **C2/3 RIJV** | 0.771 (<0.001, <0.001) | 0.971 (<0.001, <0.001) |
| **C2/3 LIJV** | 0.799 (<0.001, <0.001) | 0.900 (<0.001, <0.001) |
| **Legend:** RIJV - right internal jugular vein; LIJV - left internal jugular vein; ICC - intra-class correlation. Values are listed as ICC (p,q), with p values <0.01 considered significant. | | |
